# Characterization of Thermoluminescent Dosimeters for Neutron Dosimetry at High Altitudes

**DOI:** 10.3390/s22155721

**Published:** 2022-07-30

**Authors:** Vittoria D’Avino, Fabrizio Ambrosino, Roberto Bedogni, Abner Ivan C. Campoy, Giuseppe La Verde, Silvia Vernetto, Carlo Francesco Vigorito, Mariagabriella Pugliese

**Affiliations:** 1 Section of Naples, National Institute for Nuclear Physics (INFN), Via Cinthia, 80126 Naples, Italy; vittoria.davino@na.infn.it (V.D.); glaverde@na.infn.it (G.L.V.); 2 Department of Physics “Ettore Pancini”, University of Naples Federico II, Via Cinthia, 80126 Naples, Italy; 3 Frascati National Laboratories, National Institute of Nuclear Physics (INFN), Via Enrico Fermi 54, 00044 Frascati, Italy; roberto.bedogni@lnf.infn.it (R.B.); ivan.castro@lnf.infn.it (A.I.C.C.); 4 National Institute for Astrophysics—Astrophysical Observatory of Turin (INAF-OATO), Via Pietro Giuria 1, 10125 Torino, Italy; vernetto@to.infn.it; 5 Section of Turin, National Institute for Nuclear Physics (INFN), Via Pietro Giuria 1, 10125 Torino, Italy; carlo.vigorito@to.infn.it; 6 Department of Physics, University of Turin, Via P. Giuria 1, 10125 Turin, Italy

**Keywords:** charged particle, neutron detectors, cosmic radiation monitoring, neutron dosimetry, South Atlantic anomaly, thermoluminescent dosimeters calibration, thermal neutron facility, TLD pair

## Abstract

Neutrons constitute a significant component of the secondary cosmic rays and are one of the most important contributors to natural cosmic ray radiation background dose. The study of the cosmic ray neutrons’ contribution to the dose equivalent received by humans is an interesting and challenging task for the scientific community. In addition, international regulations demand assessing the biological risk due to radiation exposure for both workers and the general population. Because the dose rate due to cosmic radiation increases significantly with altitude, the objective of this work was to characterize the thermoluminescent dosimeter (TLDs) from the perspective of exposing them at high altitudes for longtime neutron dose monitoring. The pair of TLD-700 and TLD-600 is amply used to obtain the information on gamma and neutron dose in mixed neutron-gamma fields due to the present difference in 6Li isotope concentration. A thermoluminescence dosimeter system based on pair of TLD-600/700 was characterized to enable it for neutron dosimetry in the thermal energy range. The system was calibrated in terms of neutron ambient dose equivalent in an experimental setup using a 241Am-B radionuclide neutron source coated by a moderator material, polyethylene, creating a thermalized neutron field. Afterward, the pair of TLD-600/700 was exposed at the CERN-EU High-Energy Reference Field (CERF) facility in Geneva, which delivers a neutron field with a spectrum similar to that of secondary cosmic rays. The dosimetric system provided a dose value comparable with the calculated one demonstrating a good performance for neutron dosimetry.

## 1. Introduction

Cosmic radiation represents a significant source of natural ionizing exposure, providing an average contribution of about 13% of the total annual individual effective dose of the world population [1]. According to the origin, cosmic radiation can be divided into three components: Galactic Cosmic Rays (GCR) arising from sources outside the solar system, Solar Cosmic Rays (SCR) generated near the surface of the Sun by magnetic disturbances, and radiation from Earth’s radiation belts (Van Allen belts). At terrestrial altitudes, the detectable radiation consists of secondary particles produced by the interaction of primary Cosmic Rays (CR) on Earth’s atmosphere, consisting mainly of protons (86% of the flux) and alpha particles (~12%), with the remainder ~1% nuclei of higher atomic number, and about 1% electrons. These high-energy particles interact with oxygen and nitrogen nuclei and generate a cascade of secondary charged and neutral particles, including γ-rays, electrons, and muons, as well as protons, neutrons, pions, and lower Z nuclei. The energy spectrum extends from GeV energies [2] up to more than 10^20^ eV [3] with a power-law shape with a slope approximately constant. At the ground level, the predominant component of the secondary spectrum consists of low-LET particles (gamma rays, electrons, and muons), with a minor component of mesons, neutrons, and protons. At high altitudes, the flux increases, and the high-LET particle contribution becomes more and more significant [4,5,6]. The flux of primary CRs is modulated by the solar activity that affects the interplanetary magnetic field, according to the 11-year solar cycle (more activity, less CRs). During intense solar flares, however, energetic particles emitted by the Sun can reach Earth, increasing the amount of radiation. The majority of CRs are stopped from entering the inner atmosphere by the geomagnetic field, which acts as a natural attenuator. Due to the dipolar shape of Earth’s magnetic field, the cosmic particle flux near the poles is much higher than at the equator, in particular during solar events. In this context, the phenomena of the global geomagnetic field decreasing, especially in the Antarctic region and at high latitudes, represents an interesting field of research, as demonstrated by many works available in the literature [7,8,9,10,11]. In particular, the weakest point of the geomagnetic field of the whole planet is located in the so-called South Atlantic Anomaly (SAA) region, where the inner Van Allen belt approaches closest to Earth’s surface [12,13,14,15]. The current study was performed in the framework of the SAMADHA (South Atlantic Magnetic Anomaly Dosimetry at High Altitude) project (http://samadha.to.infn.it/ (accessed on 6 July 2022)) of the National Institute of Nuclear Physics (INFN). The project aims to perform long-term dosimetric monitoring in the SAA region at high altitude sites (laboratories of Chacaltaya in Bolivia at 5240 m a.s.l. and of Monte Famatina in Argentina at 5100 m a.s.l.) where, due to the reduced atmospheric absorption layer, the effects of space weather and geomagnetic variability could be more evident. This kind of investigation is interesting for assessing the radiological impact of cosmic radiation exposure on people involved in long-term activities (e.g., aircraft crew and astronauts) or persons living permanently at high altitudes (e.g., Andes Mountains, Tibetan Plateau, and Ethiopian Highlands). The SAMADHA project is in continuity with previous projects focusing on the dosimetry of environmental radiation for long periods at high altitudes and latitudes in the Antarctic and sub-Antarctic regions, such as HALCORD (High Altitude and Latitude Cosmic Ray Dosimetry, 2018–2019, INFN project) and CORDIAL (COsmic Ray Dosimetry In Antarctic Latitudes, 2022–2023, Italian National Program of Research in Antarctica). Previous radiation monitoring at high mountain research stations (i.e., Chacaltaya Laboratory, Testa Grigia Research Station at 3480 m a.s.l., Cervinia, Italy) has been carried out by the same groups [16,17,18,19,20,21]. The dosimetric monitoring in these areas combines scientific interest aiming to deepen the knowledge of the physical aspects of cosmic radiation with the need to meet the requirements of radiation protection recommendations for human exposure. Many studies demonstrated that aircraft crew and astronauts constitute a group of workers receiving one of the highest annual effective doses [22,23,24,25,26,27]. Because of this scientific evidence, the International Commission on Radiological Protection (ICRP) stated that the exposure to cosmic radiation of aircraft crew and frequent flyers must be managed as a planned exposure situation in order to monitor the received dose and minimize the potential risk of radio-induced effects on health [28,29,30]. In addition to workers, the assessment of the annual effective dose in the SAA region at high altitude is important for people living there permanently, who potentially receive an ambient dose equivalent to (H*(10)) higher than the limit recommended for the general public by the ICRP, equal to 1 mSv per year [28]. At high mountain altitudes, the major contributor to the radiological risk is cosmic ray-induced neutrons because of the high neutron Relative Biological Effectiveness correlated to the “radiation weighting factor” and the extended energy range of interest ranging from thermal energies to hundreds of GeV. Therefore, to the knowledge of the biologically relevant dose equivalent, information about the LET spectrum and the neutron energy spectrum is demanded since the flux-to-dose conversion factors strongly depend on neutron energy. The typical neutron energies of secondary cosmic rays vary from thermal to 10 GeV. The differential spectrum multiplied by energy shows 3 very prominent peaks at ~0.025 eV (thermal peak), 1–10 MeV (evaporation peak), and around 100 MeV (cascade peak) [16,17,31,32,33,34]. Accurate dose measurement in mixed radiation fields is one of the most challenging tasks for the research community since the radiation around the neutron source is always mixed and a combination of slow, fast, thermal neutrons, and gamma rays. It is evident that no single method or dosimeter can provide an exact and accurate response to all energy ranges of neutrons [35,36,37]. In this case, the ICRU recommends using a pair of detectors with different responses for each beam component [35]. The current work reports the results of the characterization and calibration of thermoluminescent dosimeters (TLD-600/700), using them in combination to detect the neutron thermal component of the cosmic-ray spectrum and its contribution to the total dose. For our purpose, in the framework of the SAMADHA collaboration, we first exposed the TLDs to thermal neutron irradiation facility HOTNES (HOmogeneous Thermal NEutron Source) at INFN-LNF (Frascati, Rome, Italy) for calibration and then at the CERN-EU High-Energy Reference Field (CERF) facility, which provides a mixed radiation field similar to the cosmic ray field [38]. Our investigations not only demonstrate the suitability of TLDs as radiation monitoring devices for environmental radiation dosimetry but also provide complementary information combined with those carried out by other neutron detectors. The results of testing and characterizing the TLDs allow implementation of the planned SAMADHA objectives in terms of neutron dosimetry by thermoluminescent dosimetry.

## 2. Materials and Methods

### 2.1. Thermoluminescent Dosimetric System: Dosimeters, Annealing, and Readout Signal

The passive dosimetric method based on the combined use of thermoluminescent dosimeter TLD-600 and TLD-700 (pair method) meets the ICRU recommendations for evaluating the thermal neutron dose of a mixed radiation field [33]. The physical base of detection lies in the different amounts of Lithium isotopes in their composition. The TLD-600 and TLD-700 materials are both LiF:(Mg, Ti), but TLD-700 is enriched with ^7^Li and contains a very small amount of ^6^Li, while the TLD-600 is enriched with ^6^Li to the extent of about 96%. The isotope ^6^Li exhibits a large thermal neutron absorption cross-section, which gives TLD-600 (95.62% ^6^Li, 4.38% ^7^Li) the ability to measure thermal neutron, whereas TLD-700 (0.007% ^6^Li, 99.993% ^7^Li) responds identically to TLD-600 to gamma radiation and charged particles, but it is practically insensitive to thermal neutrons [35,37,39,40,41,42,43]. Thanks to this property, determining the neutron flux is possible by subtracting the TLD-700 signal from the TLD-600 signal [44,45,46]. In this work, we used a paired set of TLD-600/700 chips from Harshaw Chemical Company (Cleveland, OH, USA). We characterized a total of 40 TLD-600 and 41 TLD-700 for neutron dosimetry. Both chips of TLD-600 and TLD-700 have a size of 3.2 mm × 3.2 mm × 0.9 mm and effective atomic number 8.2. The experimental equipment required to prepare and read the irradiated TLDs is installed at the Department of Physics of the University of Naples Federico II. Prior to each use, the dosimeters were heated using an Isotemp programmable muffle furnace (Thermo Fisher Scientific, Waltham, MA, USA) following the standard procedure of LiF:Mg, Ti material consisting in a two-temperature process: 1 h at 400 °C followed by cooldown to 100 °C for 2 h. After irradiation, the TLDs were analyzed through the reading system consisting of two components: the Harshaw 3500 manual TL reader with planchet heating system and WinREMS readout software (Thermo Scientific, Waltham, MA, USA), allowing to set the acquisition setup parameters and to monitor the TLD reading process. The TL readout signal is provided by the measure of the energy released by free electrons that return to the valence band and holes after a heating phase. Free electrons produced after absorbing enough energy from the ionization release the absorbed energy as photons. Holes can also produce photons in an analogous process. The photons are counted using a photomultiplier tube. The total number of photons detected is proportional to the number of trapped electrons and holes and, hence, is proportional to ionizing radiation. Finally, the thermoluminescence (TL) signal consists of an amount of charge (Coulomb). The reading–heating cycle takes place in two phases, i.e., a preheat zone at a temperature of 100 °C for 10 s and a reading phase of 60 s from the temperature of 100 °C up to a plateau of 400 °C with a linear heating rate of 5 °C/s. The readout takes place under a continuous nitrogen flow which aims to reduce and/or eliminate the effect of oxidation and potential triboluminescence and chemiluminescence signals not associated with the irradiation [43].

### 2.2. Ambient Dose Equivalent Calculation

The ambient dose equivalent H*(10) represents an operational quantity useful to estimate and demonstrate compliance with the specific standard for most of the measurements for radiation protection against external radiations. The ICRP recommends using H*(10) to provide a conservative estimate of the effective dose. The value of H*(10) was determined from a measurement of neutron fluence in air. We calculated the H*(10) by combining the fluence with fluence-to-dose-equivalent coefficients provided by the ICRP-74, in Table A.42 of [47], according to this expression:

(1) $$H^{*}\left(10\right)=\overset{n}{\underset{i=1}{\sum }}h_{\emptyset }\left(E_{i}\right)\emptyset \left(E_{i}\right)\;\emptyset E_{i}$$

where *n* is the total number of neutron energy bins, ∆*E_i_* is the energy width of the *i*th bin, *E_i_* is the mean energy of the *i*th bin, ∅(*E_i_*) is the neutron fluence, and $h_{\emptyset }$(*E_i_*) represent the ambient dose equivalent per unit neutron fluence (measured in pSv cm^2^), given by the fluence-to-ambient-dose-equivalent conversion coefficients [47]. We calculated the expected dose due to thermal neutrons folding the energy spectrum of the incident neutrons with the flux-to-dose conversion curve up to E = 0.5 eV. We derived the conversion factors for each energy point of the incident energy spectrum of neutron sources by linearity interpolation from values in Table A.42 of [47]. We plotted the flux-to-dose conversion factors as a function of energy on a log-log scale as recommended by ICRP [47].

## 3. Results and Discussion

### 3.1. Calibration of Thermoluminescent Dosimeters

**Figure 1:**
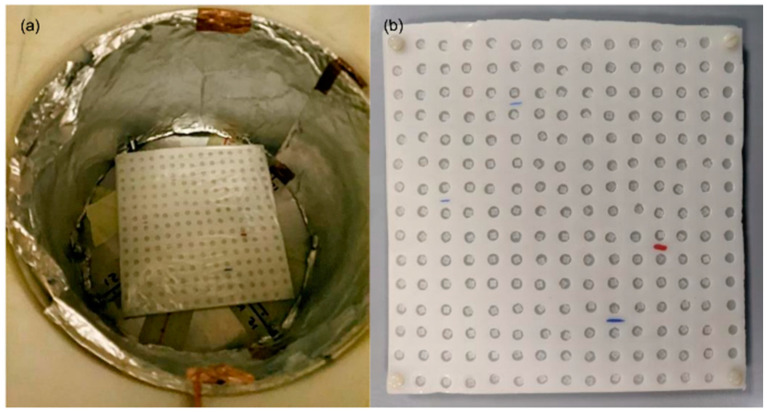
Irradiation set up at HOTNES facility: cylindrical cavity (**a**) with polyethylene support (15 × 15 grid) for thermoluminescent dosimeters (**b**).

A set of TLD-600/700 (40 TLD-600 and 41 TLD-700) uniquely coded was calibrated at the thermal neutron irradiation facility HOTNES developed at the ENEA Frascati Research Centre. HOTNES relies on a ^241^Am-B radionuclide neutron source with a nominal strength of 3.5 × 10^6^ s^−1^ located in the bottom of a large cylindrical cavity (30 cm diameter, 70 cm in height) delimited by polyethylene walls. Fast neutrons are shielded by a polyethylene shadow bar (10 cm in diameter and 20 cm in height) so that they do not reach the samples located at the upper part of the cylindrical cavity for irradiation. The irradiated volume (i.e., sample, device) receives neutrons that experienced multiple scattering events with the cylindrical cavity wall resulting in a good thermalization level (more than 90% of neutrons are thermal) neutron field. The neutron source achieves a very uniform thermal fluence rate (E < 0.4 eV) in the range 700–1000 cm^−2^s^−1^, depending on the irradiation plane chosen. Measurements of the thermal neutron fluence in the large homogeneity area HOTNES facility are available in [48], where a 2% uncertainty on the flux is reported. The angular distribution of thermal neutrons is roughly isotropic. A detailed description of the HOTNES’ design and the neutron field in the irradiation volume in terms of spatial, energy, and direction distributions are available in [49,50]. We exposed the TLDs in the central point of the +50 cm irradiation plane (reference irradiation plane) of HOTNES with the polyethylene ceiling in place. The beam ran for 65 h 4 min, recording a total fluence of 1.78 × 10^8^ n/cm^2^ corresponding to a thermal neutron fluence rate of 759 cm^−2^s^−1^. The TLDs were placed in a polyethylene support with a 15 × 15 grid designed to accommodate up to 221 samples. A group of 5 TLDs of both TLD-600 and -700 were non-exposed to radiation and used to obtain the TL measurements corresponding to the background and transport dose. Figure 1 shows the cylindrical cavity in HOTNES delimited by the polyethylene walls and support for TLDs.

**Figure 2:**
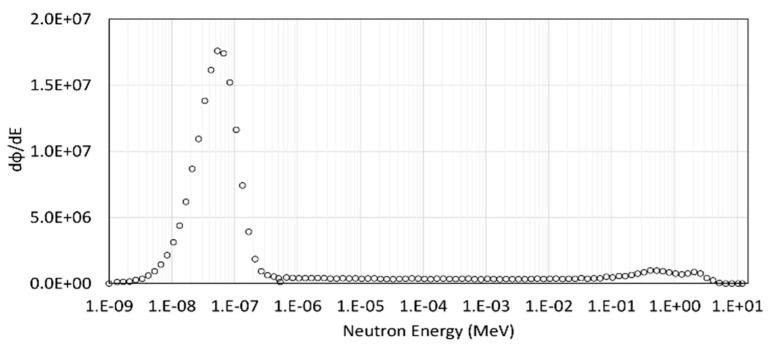
Measured neutron spectrum in HOTNES reference point with polyethylene ceiling in place. Errors are smaller than graphical symbols [49].

**Figure 3:**
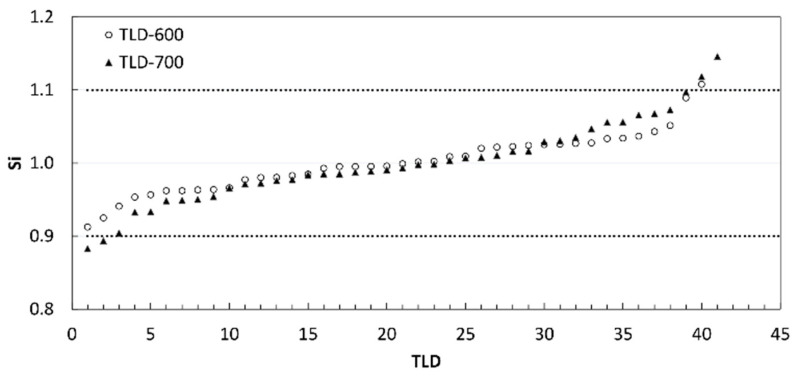
Relative intrinsic sensitivity factor for all Thermoluminescent Dosimeters (TLDs-600 and -700). The dot lines limit the range of acceptability of S_i_ [0.9–1.1].

Figure 2 shows the neutron spectral fluence measured at HOTNES during TLD irradiation. In Figure 2, we can observe that the field is highly thermalized: the fluence fraction below 0.5 eV is indeed 82%, the fluence fraction in the region 0.5 eV–0.1 MeV is 11%, and 7% from 0.1 MeV up to the maximum energy of 12 MeV. All TLDs were first characterized by determining the relative intrinsic sensitivity factor (S_i_) of each TLD to the mean of their own batch. The S_i_ expresses the response variation of each individual dosimeter around the mean. Any TLDs exhibiting a S_i_ greater than 10% were rejected, while the Si of each selected TLD was used as a correction factor when we exposed it for dosimetric use by dividing the TL response for the corresponding S_i_ value. The S_i_ for all TLDs is reported in Figure 3. S_i_ ranged between 0.91 and 1.11 for the set of TLD-600 and between 0.88 and 1.15 for the set of TLD-700. The excluded TLDs resulted in 1 TLD-600 and 4 TLD-700.

**Figure 4:**
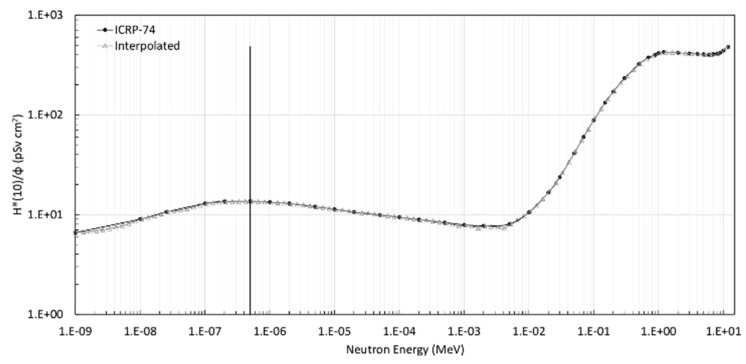
Flux-to-dose equivalent conversion coefficient on a log-log scale reported in ICRP-74 [47] and the interpolated curve in the energy values of HOTNES spectrum. The vertical solid black line indicates the energy level of 0.5 eV.

The calibration factor of the TLD-600 for thermal neutron dose (indicated as CF_600,n_) was determined as follows. We calculated the average net signal (C) of the TLD-600 and TLD-700 by subtracting the background TL provided by the corresponding non-exposed TLD group of each batch. Then, the obtained TL signal of the TLD-700, representing the gamma contribution, was subtracted from the average net TL signal of the TLD-600. The CF was calculated as the ratio between the resulting TL signal of TLD-600 and the thermal ambient dose equivalent (Sv):

(2) $${\mathrm{CF}}_{600,n}=\frac{{\mathrm{TL}}_{600,n\;}}{H^{*}{\left(10\right)}_{0.5\mathrm{eV}}}$$

where TL_600,n_ is the average neutron response reading of TLD-600 calculated as described above, H*(10)_0.5eV_ indicates the neutron thermal ambient dose equivalent. The error of TL_600,n_ was calculated as the square root of variances’ sum of the TL signal distributions of each TLD group. In order to calculate the dose absorbed by TLDs in terms of ambient dose equivalent, we folded the neutron fluence per each energy beam on the fluence to ambient dose equivalent conversion function, according to (1). Figure 4 shows the curves of fluence-to-neutron absorbed dose conversion coefficients as a function of energy as indicated in [47] and the interpolated one according to the energy bin of the HOTNES spectrum.

**Figure 5:**
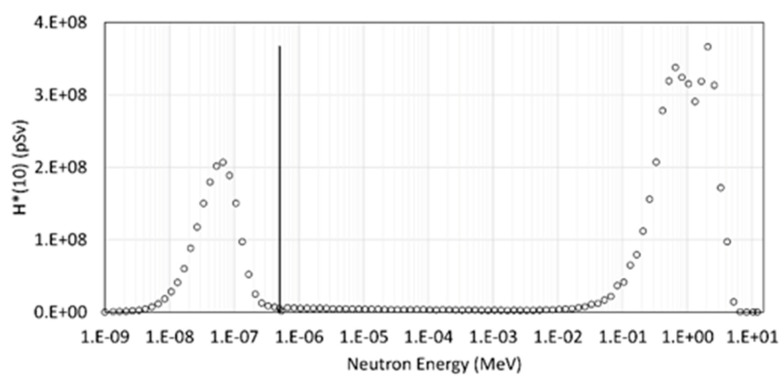
Ambient dose equivalent (H*(10)) as a function of the neutron energy. The vertical solid black line indicates the energy level of 0.5 eV.

In Figure 5, we report the contribution to the ambient dose equivalent as a function of the neutron energy. We can observe that the energy regions that provide the major dose contribution ranged between ~0.01 eV and ~0.5 eV and from ~0.05 MeV to ~4 MeV, accounting for almost the total dose with a percentage of nearly 28% and 67%, respectively. The value of the total dose is 5.76 mSv. It is interesting to observe that even if the fluence fraction of neutrons in the region 0.01–4 MeV is only ~9% of the total fluence, the contribution to the total ambient dose equivalent is about 70% of the total dose. This occurs because of the significant increase in the conversion factor toward the high energy scale, reaching 1 MeV, a value up to about 60 times higher than in the thermal region (see Figure 4).

Since we are interested in thermal neutrons, we limited the analysis to the 0–0.5 eV region. The value of the calculated neutron thermal dose equivalent is given in Table 1, together with the values of the TL response for each TLD group and the obtained CF value.

### 3.2. Irradiation Experiment: TLDs Exposure to the Neutron Field at the CERF Facility

**Figure 6:**
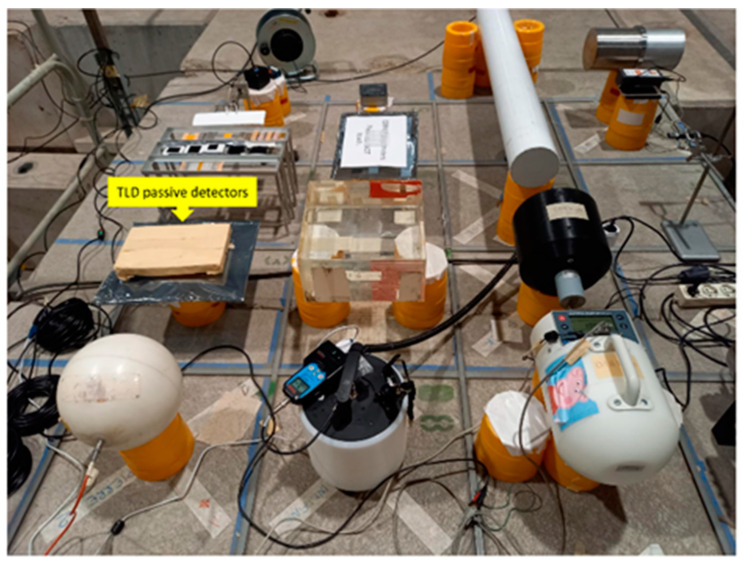
Irradiation set up at CERN-EU High Energy Reference Radiation Field (CERF) facility: the thermoluminescent dosimeters (indicated by a yellow label) were exposed simultaneously with other neutron detectors on the concrete platform.

A total of 2 groups of 37 TLD-600 and 38 TLD-700, previously characterized and selected according to the relative sensitivity value criteria (0.90 ≤ S_i_ ≤ 1.10), were used for dosimetric use at the CERF facility built at CERN in the early 1990s and installed in the North Experimental Area on the French (Prévessin) site. The CERF facility provides a high-energy workplace reference radiation field for testing, inter-comparing, and calibrating passive and active instruments employed at high-energy accelerator facilities as well as for aircraft and space dosimetry. In fact, CERF provides mixed radiation fields representative of those found outside the shielding of high-energy hadron accelerators and comparable to the cosmic ray field experienced at 10–20 km altitude. In particular, the CERF neutron beam is characterized by an energy spectrum with a shape like that of secondary cosmic ray neutrons. The CERF neutron field is produced by a meson/proton beam of 120 GeV/c interacting on a 50 cm long copper target. This beam is, in turn, produced by the primary Super Proton Synchrotron (SPS) of 400 GeV/c interacting on a 30 cm long beryllium target. Neutrons are produced in particles per SPS beam extraction (spill) about every 15 s, of variable duration around 5 s. A description of the facility’s main features, including the beam composition, exposure geometry, and values of particle spectral fluences, are carefully reported in [38]. To perform radiation exposure, we accommodated 30 TLD-600 and 31 TLD-700 into a box on the top of a concrete roof shield in one of the 16 fixed reference positions (CT3) above the interaction point, where instruments have to be installed (Figure 6). For each batch, 7 TLDs were left out of the radiation beam to carry out the average measurement of the background. The run time lasted about 16 h.

The response of individual detectors was corrected for differences in their relative sensitivity by applying the relative intrinsic sensitivity factor. In Table 2, we report the average values of the TL response for each TLD group and the neutron thermal dose equivalent obtained using the calculated CF_600,n_ reported in Table 1. The error of TL_600,n_ was calculated as the square root of variances’ sum of the TL signal distributions of each TLD group. The relative error of neutron thermal equivalent dose was calculated as the square root of the quadratic sum of relative errors of TL_600,n_ and CF^600,n^. The measured thermal neutron dose was then compared with the expected one, evaluated using the Monte Carlo simulation results given in [38], and the value of the beam intensity during the run provided to CERF users at the end of measurements. An ionization chamber (IC) allows for monitoring the beam intensity. The coefficients to derive the total neutron dose H*(10) from the IC counts are given in [38] for each reference position. These coefficients have been obtained by simulations and then checked experimentally with a LINUS rem counter, and the authors report an overall uncertainty of 11%. According to these values, the total neutron dose expected in the CT3 position integrated over the whole run time was 1.2 ± 0.1 mSv. Using the neutron spectral shape given in [38], we evaluated the thermal neutron dose, folding the spectrum with the fluence-to-dose curve [47] in the energy range up to 0.5 eV, according to (1). The estimated thermal neutron dose resulted in being a small fraction of the total dose:

(3) $${\;H}^{*}{\left(10\right)}_{0.5eV,calc}=0.014\;mSv\;$$

Assessing the uncertainty of this value is not straightforward. Besides the 11% error of the conversion coefficients from IC counts to dose, it has to be noted that the evaluation of the dose fraction due to thermal neutrons derives from a simulated spectrum. It is well known that the thermal neutron flux is highly dependent on ambient geometry, i.e., on structures and materials present in the surrounding environment that can scatter and thermalize neutrons up to large distances, and that cannot be precisely taken into account in a simulation. Moreover, the neutron spectrum given in [38] refers to the reference position CT8, and we assumed the same spectral shape in CT3, which is ~65 cm apart. From these considerations, we envisage for the value of the expected thermal dose, a global uncertainty not less than 20–30%. Considering the above uncertainties, we can conclude that the measured dose H*(10) = 0.008 ± 0.003 mSv is consistent with the expected one within the errors, thus confirming the good performance of the coupled TLD-600/700 to estimate the contribution of thermal neutrons to the total ambient equivalent dose from cosmic radiation in the context of the SAMADHA project.

## 4. Conclusions

This work, realized in the context of the SAMADHA project, allowed to check the TLD’s performance for determining the dose contribution of thermal neutrons from cosmic rays. The accuracy of the dosimetric system was provided by independent checks of TLD’s performance through different sequential phases: characterization and calibration with a ^241^Am-B neutron source in a thermalized field and subsequent exposure to flux with a typical secondary neutron cosmic ray spectrum at the CERF facility. The results represent a first step for further implementation of the project objectives, including the dosimeters exposure at high altitudes for a long period. The dosimetric information provided by TLDs in the low energy neutron spectrum will be combined with those provided by neutron dosimeters sensitive over other energy ranges of the cosmic ray spectrum, reaching a complete knowledge of the received dose at high altitude in the perspective of neutron monitoring for radiological protection. The pair of TLD-700 and TLD-600 can be appropriate for neutron dosimetry at high mountain altitudes if the calibration is performed with a well-known neutron spectrum and applied for a similar energy spectrum. In addition, accurate response is provided after correction of the TLD’s response by an individual correction factor.

## Figures and Tables

**Table 1:** Measured signal of thermoluminescent dosimeters (TLD-600 and TLD-700), neutron thermal dose equivalent (H*(10)_0.5eV_), and calibration factor for TLD-600.

	TLD-600	TLD-700
TL exposure (nC)	520 ± 18	8.6 ± 0.4
TL background (nC)	3.6 ± 0.2	3.5 ± 0.1
TL_600,n_(nC)	511 ± 18	
H*(10)_0.5eV_ (mSv)	**1.67 ± 0.03**	
**CF_600,n_ (nC/mSv)**	**306 ± 12**	

**Table 2:** Measured signal of thermoluminescent dosimeters (TLD-600 and TLD-700) and neutron thermal dose equivalent (H*(10)_0.5eV_).

	TLD-600	TLD-700
TL exposure (nC)	6.8 ± 0.4	4.7 ± 0.6
TL background (nC)	2.4 ± 0.2	2.67 ± 0.16
TL_600,n_ (nC)	2.4 ± 0.8	
H*(10)_0.5eV_ (mSv)	**0.008 ± 0.003**	

## Data Availability

Not applicable.
